# Healthcare professionals’ perspectives of the management of people with palliative care needs in the emergency department of a UK hospital

**DOI:** 10.1186/s12904-023-01248-8

**Published:** 2023-09-06

**Authors:** Jane Sausman, Azra Arif, Annie Young, John MacArtney, Cara Bailey, Jaimini Rajani, Rebecca Burt

**Affiliations:** 1 University Hospitals Coventry and Warwickshire, Coventry, UK; 2 Warwick Medical School, University of Warwick, Coventry, UK; 3 Institute of Clinical Sciences, University of Birmingham, Birmingham, UK

**Keywords:** People with palliative care needs, Admission to hospital, Through emergency department (ED), ED healthcare professional perpectives, Need coordinated service

## Abstract

**Background:**

The Emergency Department (ED) is not always the optimal place for people with palliative care needs but is the most common route for treatment when urgent care is sought. The aim of this study,’’REasons for PalLIative Care Admissions (REPLICA)’ was to explore the perspectives of ED healthcare professionals of hospital admission or discharge via ED for palliative care patients.

**Methods:**

This is a sequential mixed methods study comprising (i) quantitative descriptive analysis of Hospital Episode Statistics (HES) of palliative care patients (code Z51.5) who were admitted through ED in a West Midlands Hospital and for the rest of England; (ii) in-depth semi-structured interviews with 17 ED staff which were analysed using thematic content analysis.

**Results:**

Over the four years (2013–2017), 430,116 people admitted through ED were identified with a Z51.5 diagnosis code, 0.6% (n = 2736) of whom were from the West Midlands Hospital. The most common reasons for palliative care patients’ admission to hospitals across England were for care of chronic kidney disease, cancers and urinary tract infections. Five themes were elicited from the qualitative analysis: (1) *Providing palliative care in ED is challenging*, due to factors including lack of training in palliative care and the unsuitable environment. (2) *Patients go to ED due to challenges in community management* such as inappropriate referrals and no care plan in place. (3) *Health system influences admission and discharge decisions*, including bed availability and being unable to set up community services out-of-hours. (4) *Discussion with patient about treatment and end of life care needs to be outside of ED* whilst the patient is still well enough to express their wishes. (5) *Improving services for patients with palliative care needs.* Recommendations include short training sessions for ED staff and accessing palliative care professionals 24/7.

**Conclusions:**

A large number of palliative care patients visit ED and are admitted to hospital for care; there is an urgent need to prevent patients attending the hospital through the establishment of a coordinated and dedicated service to support palliative care patients in the community.

**Supplementary Information:**

The online version contains supplementary material available at 10.1186/s12904-023-01248-8.

## Introduction

The UK population is ageing, with more people living longer often with complex long-term conditions leading to a significant rise in those with palliative care needs [1]. Prior to the COVID-19 pandemic, the number of emergency medical admissions (EMA) in England rose 28% between 2011 and 2019, resulting in a crisis of care in the NHS and causing considerable pressure on health and social care capacity [2]. Post-pandemic, EMA are currently back to around pre-pandemic levels [3]. Reducing the number of emergency admissions continues to be a central aim of health policy in the UK, alongside the provision of innovative services, often in the community [4]. People with palliative care needs, including end of life (EOL) care, frequently receive suboptimal management in hospital and many admissions are potentially avoidable [5]. Healthcare professionals also often deem EMA as ‘inappropriate’ and ‘potentially avoidable’ for this population [6].

The primary reasons for presentation at The Emergency Department (ED) pre-pandemic for people with palliative care needs were respiratory distress, pain and gastrointestinal symptoms [7]. Often ED is the only ‘viable option’ available to patients with exacerbation of symptoms [8] as patients and carers may struggle to access community support or lack knowledge on alternative sources of care [9]. Early palliative care and enhanced supportive care improve outcomes for people with advanced cancer [10–12] and yet we see ED as the most frequent place where urgent care is sought, perhaps indicative of paucity of a shared, planned and coordinated pathway of palliative care.

The Emergency Department has been found to be pivotal in determining patients’ goals of care, discussing prognosis and disease trajectory and initiating palliative care [13, 14]; ED is where many palliative care patients go for support. However, the suitability of ED for these purposes must be questioned since it is a less than ideal environment for the delivery of palliative care and may cause distress to patients and carers [15]. Nevertheless, pragmatic steps, such as the use of ‘palliative care champions’ among ED workforce and the introduction of palliative care competencies into the training programme in emergency medicine, [16] may help to mitigate the challenges of caring for people with palliative care needs (‘palliative care patients’) in ED.

Research into the development of emergency palliative care services has seldom included input from healthcare professionals [17]. The decision-making processes of ED healthcare professionals assessing and caring for palliative care patients and the factors that may influence avoidable hospital admissions for this patient population will reveal whether there is the potential to modify existing practices to improve care locally.

The aim of this study entitled ‘REasons for PalLIative Care Admissions (REPLICA)’, carried out pre-pandemic, was to explore the reasons why palliative care patients were being admitted to ED and make recommendations to support ED clinicians delivering palliative care in ED. To achieve this, we first quantitively explored the number of people being admitted to hospital through the ED with palliative care needs in a West Midlands (WM) Hospital and in the rest of England.

## Methods

### Design

This is a sequential mixed methods study comprising a quantitative analysis of Hospital Episode Statistics (HES) and in-depth interviews with Emergency Department staff.

### Part 1 quantitative

#### Dataset description

In Part 1 of the study, we studied the number of admissions of patients with palliative care needs through Emergency Departments using the National Health Service Hospital Episode Statistics (HES) database. An application (DARS-NIC-113,611-X2Y3H) to obtain and use the data was made to NHS Digital, UK. The HES database comprises anonymised details of individual patient admissions recorded by clinicians and coders at English hospitals. A HES ID allows the patient to be tracked through diagnosis, procedures to discharge or death. Patient ‘episodes’ are coded at discharge with the International Classification of Diseases (ICD) palliative code of Z51.5 along with any other diagnosis made during the episode; comorbidities are also added to the diagnosis coding string. Procedures undertaken during the episode are coded with OPCS Classification of Interventions and Procedures codes [18]. Codes can only be applied if there is clear documentation of the diagnosis and procedure in the patient notes.

Data for patients coded Z51.5 (palliative care) admitted via ED for four years between March 31st 2013 and April 1st 2017 for a West Midlands Hospital and all other hospitals in England were analysed. The available datasets included diagnoses, comorbidities, admission medical speciality, gender and age range.

At the West Midlands hospital, referrals to the palliative care team are made internally by emergency or inpatient ward staff. The palliative care team review referrals against their criteria and if deemed appropriate, the patient is registered under the palliative service. Post discharge or death the inpatient admission is reviewed by the clinical coding team where they will apply the Z51.5 code based on the documentation in the clinical notes. This process may vary across England. Palliative care team members make multiple patient and carer (where appropriate) ‘contacts’ to assess, implement and review patient care in hospital and, occasionally, post-discharge.

#### Analysis

The data were analysed using SAS software version 6.3. Due to the high number of records from HES, data were loaded into SQL tables and queries written to identify the agreed key performance indicators. Data were extracted into Excel for charts. Descriptive statistics were used, with percentages calculated for two variables, namely reasons for admission and clinical specialties to enable comparisons between the West Midlands Hospital and other hospital providers across England (‘rest of England’). All variables were normally distributed except for length of hospital stay which is presented as median values.

### Part 2 qualitative

#### Recruitment

Purposive sampling [19] was used to identify 17 participants. The participants were qualified medical and nursing staff working in the West Midlands hospital ED, with some experience of caring for those with palliative and EOL care needs. Potential participants who met the inclusion criteria were identified and sent an email, with the participant information sheet, by the research sister working in ED. They were asked to contact the chief investigator directly if interested in participating.

#### Data collection

A semi-structured interview guide was developed for this study comprising largely of open-ended questions, based on aim of the study and informed by the literature [20] [supplementary file 1]. The semi-structured format enables interviewers to follow-up responses and, when required, uses probes to elicit a more in-depth response [21]. Based on the participants’ experiences of treating palliative care patients in ED, the areas discussed were: understanding of the term ‘palliative care’ and views on treating palliative care in ED; why patients with palliative care needs attend ED; palliative care processes in ED including admission and discharge and what could be done differently to improve future treatment of patients with palliative care needs?

The interviews (duration 25–60 min) were conducted face-to-face in a private room in the hospital, outside of ED, between September and December 2019 by two members of the research team (AA and RB). The interviews were recorded and transcribed verbatim.

#### Analysis

The data were analysed using Thematic Content Analysis [22]. The researchers who interviewed participants undertook the initial coding. After reading and re-reading the transcripts to enable familiarisation with the data, short phrases of text were identified and labelled [23]. The first three transcripts were independently coded by both researchers, as described by Elliot [24]. When the coding and labels were compared, it was found, by and large, that there was good agreement. The coding of the first three transcripts was also discussed with the rest of the research team and, where necessary, codes were modified, with the changes used to inform subsequent coding. A list of codes was developed; further codes were added when the remaining transcripts were coded. Other members of the research team were involved in forming the categories and themes by grouping similar codes together and renaming where appropriate. The themes and categories were again reviewed in relation to the transcripts to form the final five themes and categories.

#### Ethical considerations

The Research and Development (R&D) department at the West Midlands Hospital, in line with the Governance Arrangements for Research Ethics Committees (GAfREC), reviewed the protocol and confirmed that REC or Health Research Authority (HRA) review was not required because the participants were staff, by virtue of their professional work, rather than NHS patients or service users.

Participants had the opportunity to ask questions before agreeing to take part and signed a consent form prior to being interviewed. The interviewers were mindful that the discussion could have been upsetting for some participants, especially when recalling specific cases, in which case the interview would have been paused or terminated, depending on the wishes of the participant. At the start of the interviews, participants were advised that they could choose not to answer questions and terminate the interview at any time without giving a reason. Participants were identified by codes rather than names in the transcripts and report.

## Results

### Part 1 – Quantitative hospital episodes statistics – palliative care

Over the four years (2013–2017), 430,116 people admitted through EDs were identified with a Z51.5 code, 0.6% (n = 2736) of whom were from the West Midlands Hospital (Table 1). Year by year admission data are available on request.

**Table 1:** Number of People admitted 2013–2017 with Z51.5 code

	Male	Female	Not stated	Unclassified	Total
Rest of England	213,327	214,038	2	13	**427,380**
WM Hospital	1,398	1,338	0	0	**2,736**
**Total**	**214,725**	**215.376**	**2**	**13**	**430,116**

Key: WM West Midlands

Rest of England = Figures for England minus WM Hospital

**Fig. 1:**
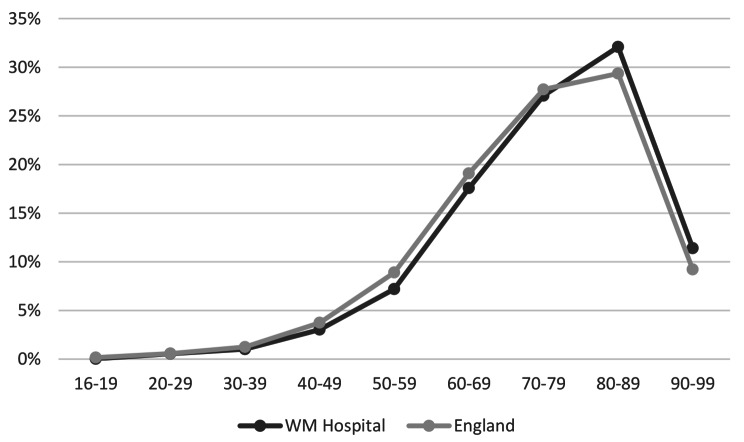
Age Groups of People in Rest of England and WM Hospital with Z51.5 code

The age group of this population on their first admission increased over the years and peaked at 80–89 years [Fig. 1]. Ethnic origin of palliative care patients was similar between the rest of England and the inner-city West Midlands Hospital with the exception of ‘Asian British – Indian’ people (1% in the rest of England; 4% in the WM Hospital [data on request]).

The most common reasons for admission to hospital across England for people allocated the Z51.5 code over the four years were: care of people with chronic kidney disease, various cancers, in particular, in the metastatic stage, urinary tract infection (UTI) and lobar pneumonia (Table 2). This maps onto the most common admission specialities, for example, to General Medicine, Oncology/Haematology, Nephrology and Thoracic Medicine (Table 3).

**Table 2:** Reasons for Admissions for People allocated Z51.5 Code: Rest of England 2013–2017

Code	Description	Rest of England n (% total admissions – people allocated Z51.5 code)
N185	Chronic kidney disease, stage 5	169,413 (3.48%)
C900	Multiple myeloma	94,415 (1.94%)
C509	Malignant neoplasm of breast	76,841 (1.58%)
C349	Malignant neoplasm of bronchus and lung	67,400 (1.38%)
J181	Lobar pneumonia	62,667 (1.29%)
C795	Secondary malignant neoplasm of bone and bone marrow	58,894 (1.21%)
C920	Acute myeloblastic leukaemia	51,571 (1.06%)
C56X	Malignant neoplasm of ovary	52,548 (1.08%)
N390	Urinary tract infection	52,114 (1.07%)
N189	Chronic kidney disease, unspecified	49,974 (1.03%)

**Table 3:** Most common medical speciality for hospital admission for people allocated Z51.5 code: England 2013–2017

Code	Description	Rest of England (% total admission specialities)
300	General Medicine	649,965 (13.34%)
370	Medical Oncology	538,311 (11.05%)
800	Clinical Oncology	456,721 (9.37%)
303	Clinical Haematology	443,853 (9.11%)
361	Nephrology	257,024 (5.27%)
430	Geriatrics	217,531 (4.46%)
340	Thoracic Medicine	191,445 (3.93%)
301	Gastroenterology	168,083 (3.45%)
100	General Surgery	153,583 (3.15%)
101	Urology	113,070 (2.32%)

Although the reasons for admission and specialities were broadly similar in the West Midlands Hospital, adjustment and management of vascular access device was also found to be a common reason for admission (Table 4) and featured in the admission specialities (Table 5).

**Table 4:** Reasons for Admissions for People allocated Z51.5 Code: WM Hospital 2013–2017

Code	Description	WM Hospital n (% total admissions – people allocated Z51.5)
C900	Multiple myeloma	1,264 (3.30%)
Z452	Adjustment and management of vascular access device	939 (2.45%)
C20X	Malignant neoplasm of rectum	670 (1.75%)
N390	Urinary tract infection	600 (1.57%)
C795	Secondary malignant neoplasm of bone and bone marrow	575 (1.50%)
C56X	Malignant neoplasm of ovary	565 (1.48%)
Z451	Adjustment and management of infusion pump	544 (1.42%)
J181	Lobar pneumonia	488 (1.27%)
C509	Malignant neoplasm of breast	470 (1.23%)
C920	Acute myeloblastic leukaemia	419 (1.09%)

**Table 5:** Most common medical speciality for hospital admission: WM Hospital 2013–2017

Code	Description	WM Hospital
800	Clinical Oncology	9,444 (24.67%)
303	Clinical Haematology	2,753 (7.29%)
430	Geriatrics	2,367 (6.18%)
300	General Medicine	2,214 (5.78%)
340	Thoracic Medicine	1,859 (4.86%)
301	Gastroenterology	1,693 (4.42%)
100	General Surgery	755 (1.97%)
180	Accident & Emergency	681 (1.78%)
361	Nephrology	549 (1.43%)
320	Cardiology	532 (1.39%)

Interestingly, the average length of stay in England increased over the four years from 6.8 days in 2013/2014 to 8.3 days in 2016/2017 (Table 6).

**Table 6:** Length of Stay for People with Z51.5 Code: England and WM Hospital 2013–2017 combined

Year	Median length of stay in days Rest of England	Median length of stay in days WM Hospital
2013-14	6.80	6.45
2014-15	7.08	6.97
2015-16	7.42	7.71
2016-17	8.30	9.06

Despite the limitations with the coding procedures discussed below, the HES data on people assigned the palliative care code show that large numbers of people are admitted to hospital via ED each year with palliative care needs. These raw baseline data led us to the second part of the study, to explore the perspectives of healthcare professionals in ED of the admission to, or discharge from, hospital via ED for palliative care patients.

### Part 2 Qualitative - interviews

#### Characteristics of participants

The participants’ characteristics are summarised in Table 7. The age range of the participants was 26–51 years. Ten had worked in the Emergency Department for more than five years. The themes and categories derived from the analysis are shown in Table 8.

**Table 7:** Participant characteristics

Characteristic	Description	Number
Gender	Male Female	6 11
Position	Consultant doctor Other doctor Advanced nurse Senior nurse	3 6 6 2
Years working in emergency medicine	0–5 6–10 > 10	7 6 4

**Table 8:** Themes and categories

Themes	Categories
Providing palliative and EOL care in the ED is challenging	• Medical conditions associated with needing palliative care. • Recognition of special care needs of EOL patients; need to treat patient and care for family. • Challenges due to staff having expertise in emergency medicine rather than treating patients with palliative care needs. • Inappropriate physical environment.
Patients go to the ED due to challenges in community management	• Decision, often inappropriate, by GP, nursing home/hospice staff or paramedics. • Should be cared for at home by community teams/poor management in the community. • Patient/family unable to see GP/contact community services, especially out of hours. • Family cannot cope with patient struggling at home.
Health system influences admission and discharge decisions	• Patient/family’s wishes (may be expressed in ReSPECT/ACP). • It is easier to admit than discharge. • Medical needs of patient changing. • Availability of hospital and community support; linked to time of day. • Practical reasons e.g. no beds, may admit if long wait time, including above the four hour requirement.
Discussion with patient about treatment and EOL care needs to be outside of the ED	• Knowing patients’ wishes about treatment through a ReSPECT form or ACP makes it easier for medical staff. • If no record of a patient’s wishes, may be given all investigations and CPR. • ED consultants routinely discuss EOL treatment with patients and complete the ReSPECT form. • ED is not the right place to have a conversation about dying; needs to at an earlier stage, outside the ED. • There may be a conflict between the patient’s verses the family’s wishes.
Improving services for patients with palliative care needs	• Need to be able to access staff with expertise/responsibility for palliative care. • Regular short training/information sessions for ED staff. • Discussions with patients and completion of ReSPECT or ACP outside of ED, before EOL care is needed. • Information to patients/families on services/support available in the community.

### Theme 1: providing palliative and EOL care in the ED is challenging

When asked about which conditions they associated with the term palliative care, nearly all participants mentioned cancer or oncology. The majority also referred to other chronic conditions such as COPD, stroke or dementia, although a few felt that patients with other conditions did not receive the level of community support given to cancer patients. Most participants’ definition of palliative care was orientated around there being no further curative treatment options available, with the emphasis being on symptom control. It was acknowledged by some that patients may live for several years after treatment had stopped.

So I would say that palliative care means the management of symptoms to do with a long term life limiting illness, whereas end of life care I would consider as your last, sort of, few weeks, few days, few hours, and how we manage that and make them, make you comfortable, make you, and respect your wishes ….

(P11, advanced nurse)

Within this theme, the general challenges of caring for patients with palliative care needs in an ED are addressed. Several participants acknowledged that it could be difficult to identify which patients had palliative care needs. This was in part due to challenges, especially when busy, in locating information about a patient’s medical history, for example from the Clinical Results Reporting System (CRRS) or the GP summary care records. None of the participants were aware of any tools which can be used in ED to identify patients with palliative care needs. When shown the Supportive & Palliative Care Indicators Tool (SPICT) [25], three felt that it could be useful, although others expressed reservations due to the high workload for staff in ED.

Challenges identified in developing clinical skills included the six-monthly rotation of junior doctors and the high turnover of staff. None of the participants had attended formal training on palliative and EOL care. Several had attended ad hoc short training sessions, although the content was not always applicable to ED. Over half of those interviewed referred to learning about palliative and EOL care through on-the-job training by consultants, which was viewed as a valuable way of enhancing clinical skills. It was pointed out that there are regular discussions between colleagues about cases and that there is always someone to approach for advice. A few participants referred to having expertise across a wide range of conditions or symptoms seen in ED rather than being specialists in longer term, chronic conditions:

*“ … we can do it [hold a discussion with a patient about their respiratory condition], but it can be perceived as, you know, we’re stepping out of our role. And to be fair, to, to an extent, it is because, you know, we’re, we know little bits about a variety of vast subjects, but we’re not specialist in one single subject.”* (P5, doctor).

P1 (advanced nurse) explained how she had been trained to save lives or provide care when death is unexpected.

…well, I feel that we’re not the right people for the job; we’ve been trained to try and save people’s lives, so when it comes to an end of life there’s palliative nurses out there who specialise in it, there’s end of life nurses, there’s geriatric nurses that have had way more training than we have in this situation. So we have the sudden death experience but we don’t have the palliative, where the family know and it’s going to be a drawn-out process.

When considering the needs of patients with palliative care needs attending ED, the majority initially discussed EOL care. Responses demonstrated the desire to show compassion and to make patients as comfortable as possible and ensure the best death possible. Two participants referred to it being a privilege to care for someone at the end of their life. Several mentioned that patients with palliative care needs, particularly if near their EOL, were given priority in terms of being seen and finding a private cubicle within ED. The need to care for the family, as well as the patient, was highlighted by several participants.

So it’s important because it’s the last thing I can do for someone; inspect them, give them a good death, and because you treat not just the patient, quite a lot of what I do in the emergency department for that batch of patients, is I treat their family.

(P17, doctor)

Although doing their upmost to meet patients’ needs, all participants felt that ED was not the appropriate place to treat patients with palliative care needs, especially when towards the end of life, unless they had symptoms which could not be alleviated elsewhere. The physical environment in ED was felt to be particularly unsuitable. Adjectives used to describe the ED included ‘noisy’, ‘busy’, ‘crowded’ ‘impersonal’, ‘hellish’ and ‘awful’.

The environment is just horrible. So if somebody’s, you know, the main aim of end-of-life palliative care is, I think, comfort. And A&E is not the place for comfort.

(P3, advanced nurse)

Waiting times could be up to 12 h and, if very busy, there was little time to talk to the patient and the family. When crowded, there was no area within ED to hold a private conversation leading some participants to take families elsewhere:

And sometimes in a corridor and sometimes in the room where we take patients to go to the toilet [using a commode] ‘cause it’s the only place that I can go ….

(P2, consultant)

Feelings of frustration were expressed about not being able to give more time and effort and provide better care.

### Theme 2: patients go to the ED due to challenges in community management

The most common medical reasons for patients with palliative care needs attending ED were identified as needing pain control, medication running out, or medical needs having changed. However, the overriding issue raised by participants within this theme was the need for community services to manage patients at home.

*“ … I’ve met a fair few people who don’t want to die in a hospital, don’t want to come into a hospital, and if they could meet a GP or someone who could prescribe in the community, who had the spare time to go and sort out their issues, there would be no reason for them ever to get hooked into a four hour wait followed by sitting on a trolley for hours, followed by an admission that lasts three days that they never needed in the first place.”* (P5, doctor).

P6 (consultant) similarly felt that patients with palliative care needs present at ED due to a “failure of the system”:

*“But I think sometimes we see it, it’s I suppose a generalisation, but you see it as a failure of the system that they’ve ended up needing to come to the emergency department for their care. And you feel that somehow that if systems and processes had been there or they’d been identified earlier that you might have managed to make those patients more comfortable or have plans in place which means that they don’t need to come and wait four hours in ED.”*

Several participants felt that no care plan to prevent admission to ED, or lack of understanding of patients’ needs, frequently resulted in inappropriate referrals to ED.

Participant 17 (doctor) felt that ED was often viewed as a universal safety net:

…we’re a safety net, they will get seen. Yeah, but that, I think that’s it, you get, end up just being the universal safety net. You know, if someone’s not sure, the family are complaining, you’re worried about XYZ, send them, it’s an easy decision; the decision’s off your plate.

However, it was acknowledged that a serious deterioration in the medical condition of patients, can result in there being.

*“… only one option to ring 999”* (P15, advanced nurse).

The fact that paramedics must make difficult decisions about whether to take patients to hospital or leave them at home for their last few hours or days was raised by several participants. P15 went on to say:

And that’s the sad part about it is that because of belt and braces and things like that, you know, you are not allowed to die, once you’re under the care of an emergency service. So the ambulance service are not good at saying, “Well, there’s nothing for us to do here. We’ll just sit, put the kettle on and talk to you and wait.

Patients often went to ED because they or their family were unable to see the GP or contact community services, sometimes due to not having the contact details, and did not know where else to go. This is often during the night or at weekends. The role of the family in requesting that the patient be taken to hospital was highlighted, even though the patient may prefer to be treated and die at home. This was attributed to families finding it difficult to cope with seeing their family and friends in distress or dying.

*“Or the family are struggling to see their relatives like that [in pain] and they thought they would be able to cope, so they’ve brought them in ‘cause they’re panicking.”* (P1, advanced nurse).

### Theme 3: health system influences admission and discharge decisions

All patients arriving at ED are admitted and assessed by a triage nurse. This theme encompasses the various factors which influence the decision to discharge a patient home from t ED or admit as an in-patient.

A third of participants mentioned the need to consider the wishes of the patient or family, which may be expressed in writing, for example in a Recommended Summary Plan for Emergency Care and Treatment (ReSPECT) form [26] or discussed with clinicians in ED.

A few considered that the requirement that patients should not have to wait in ED for longer than four hours could influence the decision to admit a patient.

So last night [we had] 26 on the corridor, really sick patients, need to get them into cubicles. If there are spaces within the medical teams within the organisation and I can’t solve the problem for a patient in the, in the first five or six hours and it should really be within the first four hours, but if I can’t get resolution in that period of time ….

(P2, consultant)

Although it was acknowledged that patients may need to stay in hospital due to their medical needs, the decision to admit or discharge a patient was largely determined by practical considerations. Patients are sent home if there are no beds available unless the patient cannot manage at home, in which case they will need to wait for a bed. However, if there is a long waiting time in ED or it is outside normal working hours, which makes it impossible to set up community support services, patients were more likely to be admitted. The time of day was repeatedly brought up by participants as being an important factor when deciding whether to admit or discharge patients from ED. P14 (doctor) described some of the challenges faced by clinicians when patients attend ED out of hours:

*That there isn’t anybody on site that can come, come and review the patient and to set up sort of the things that that patient may need. So, whether that be support at home or… and we only have a limited time in A&E and we have a lot of time pressures, and to sort that sort of stuff out is often maybe lot, a lot of phone calls and it’s perceived as being easier to just admit them and then that can be done during the day.*

Several participants referred to the benefits of keeping patients in the observation ward or admitting to the Acute Medicine department for a short stay to enable symptom control, the palliative care team to be contacted and support to be set up in the community. Patients are only discharged from hospital once a management plan is in place.

### Theme 4: Discussion with patient about treatment and EOL care needs to be outside of the ED

One third of participants referred to importance of patients having a ReSPECT form or Advanced Care Plan (ACP) to enable a patient’s wishes regarding treatment and their preferred place of care to be expressed. Participant 9 (doctor) explained the value of having a ReSPECT form:

… that means we don’t have to search through the notes, search through the reams and reams of clinic letters to work out if someone has had that discussion, and if so, what the patient wants.

Although some participants felt that, in the absence of a formal expression of wishes, it was important to discuss treatment options with patients, several mentioned that without a ReSPECT form or ACP, some doctors will arrange all investigations, which may be invasive and uncomfortable, and give CPR, even if the patient is near the end of their life. Participant 15 explained that when there is no ReSPECT form staff feel “*duty bound*” to give treatment even if they know that palliative care is more appropriate. However, a few problems were identified with making decisions about care even with a ReSPECT form. Sometimes the forms lack sufficient detail to determine the ‘ceiling of care’ or whether patients prefer to be at home. Several participants had experienced a disconnect between what was on the form and the expectations and wishes of the family members, often because they had not been present when the form was completed:

*“I couldn’t begin to describe how common that is in my working day to go to see a patient whose family have no idea that this patient is dying.”* (P2, consultant).

The importance of having ‘that conversation’ about dying was repeatedly raised by participants. They emphasised the necessity for family members to have discussions with each other about dying and their wishes, as well as the need for clinicians to approach this topic with their patients and complete a ReSPECT form or ACP whilst the patient is well enough to be able to consider treatment options and express their preferences.

*“I know it’s, it’s difficult and it’s idealistic, but having that planning of “What’s going to happen in your condition, do you actually want to be admitted in the hospital or not? Because the treatment’s going to be this and the outcome is going to be this anyway.” But having that discussion done at an earlier point …”* (P4, doctor).

Although ED consultants routinely discuss EOL treatment with patients and complete the ReSPECT form, the view ED was not the right place to have this discussion was widely expressed.

*“… most of my colleagues feel the same way, there’s probably a, what on the face of it and what may be from their point of view a very good reason for not having those conversations, I just, I am certain that the right place to do it is not when you’re already sick at two in the morning after you’ve sat on a corridor for four hours when you may actually be dying…”* (P5, doctor).

### Theme 5: improving services for patients with palliative care needs

Participants made a number of suggestions for improving the care they are able to give to patients with palliative and EOL care needs. Almost all participants identified the need to be able to access staff with expertise in palliative care at any time of day, including at weekends. Several suggestions were made to accomplish this. There could be a link nurse and/or doctor working in ED who will undertake other duties when not seeing a palliative care patient or offering advice on their care.

Participants felt that the palliative care team should be able to offer assistance in the hospital 24/7, as is the case for stroke patients. It was also proposed that the palliative care advice line should be available 24/7.

*“Well, it would be lovely if the Palliative Care team advice line was 24/7. It would be really useful to, and have that as the same number 24/7 so that you weren’t swapping between a hospital bleep and a, a phone number outside those times. So, having people who actively know what the current hospice bed state’s like, actively know what community services, what, where they’re full, what they’re like, may know the patients themselves …”* (P6, consultant).

Other suggestions included a turnaround service for patients with palliative care needs, as there is for infection and falls, a designated area in ED for treating patients with palliative care needs, especially when near the EOL, which could be sound proofed as it is in the Paediatric Outpatients Department, and short drop-in sessions in hospices.

From the interviews, it is evident that there are other changes in processes which would positively impact upon the care given to patients with palliative care needs. Increasing the proportion of patients admitted to ED having a completed ReSPECT form or ACP would help clinicians to consider patients’ wishes regarding their treatment, including at the EOL. Patients and carers need information on services and support available in the community and how to contact services, including out-of-hours. Several participants suggested regular, short training sessions for new staff and as a refresher for experienced staff on services and alternative pathways, processes, caring for patients with palliative and EOL care needs and on the resources available.

*“I think education is always helpful. I don’t think I’ve had any teaching about palliative as I said since I was in medical school or maybe foundation … A better understanding of actually what we can do in A&E ‘cause as I say, I’m probably completely wrong when saying that I don’t feel that I can call anyone out of hours.”* (P14, doctor).

Sessions need to be designed to take account of the situation in ED. Others working in the community, such as GPs and nurses may also benefit from training sessions on the different pathways and providing care for patients with palliative care needs including at the end of life.

## Discussion

The first stage of this study was the analysis of the palliative care HES data for the UK and the study site hospital, over a four-year period between 2013 and 2017. On average, over 107,000 palliative care patients, mostly elderly, identified by the code Z51.5 1 were admitted through ED per year in England during this period. Over the four-year period, there was an increase in this population of patients admitted through ED until the last year analysed, when it fell (2016-17). The reasons for this fall in patients admitted are unknown. These palliative care patients were mostly admitted for cancer care but also for concerns due to chronic kidney disease stage 5 and urinary tract infections. Interestingly, UTI has been found to be one of the most encountered infections in palliative care units [27, 28]. The cause of UTI may be due to many factors including an Indwelling urinary catheter. What we see is probably a large underestimation of need, because of the limitations of the Z51.5 coding system and the underutilisation of this system (see limitations).

One bonus on exploring the HES data locally and comparing nationally, is that apparent local issues can be fed back to the local teams for service improvement e.g. palliative care patients being admitted for central venous catheter problems.

These large raw numbers showing reasons for admission and various medical specialities provide an indication of the extent of the challenge. It was therefore decided to examine why palliative care patients present at ED and the processes which lead to admission or discharge in greater depth in the second stage of the study. The Emergency Department staff from the study site hospital were interviewed about their perspectives on caring for patients with palliative and EOL care needs presenting at ED.

All participants wanted to provide the best possible care for palliative and EOL care patients and their families, although a few felt frustrated about their lack of training and experience in the palliative care field. It was felt that, provided they have the appropriate equipment and drugs, doctors in ED can effectively manage the symptoms palliative and EOL patients present with, such as pain and a deterioration in a patient’s medical condition. This was in contrast to the findings from a qualitative study by Bayuo et al. in which data were also collected using in-depth interviews with emergency department staff [29]. Participants experienced feelings of failure and helplessness when treating patients requiring EOL care. The findings from the study by Cooper et al., that lack of time and resources often lead to patients with palliative care needs being a low priority [16], was similarly not echoed by participants in our study. The opposite view was expressed in REPLICA, that priority was given to patients with palliative care needs. For those near their EOL, the need to “give them a good death” and to care for the family was highlighted by several participants.

Nonetheless, all participants felt that ED was generally not the most appropriate place to treat palliative and EOL care patients, which agrees with the findings of several other studies [30–33]. In particular, the busy, noisy environment, as well as lack of time, were highlighted as barriers to providing the care needed. However, several participants were able to describe cases where ED had been the best place for EOL care for that patient, for example due to being able to provide the most effective treatment quickly or the only relative present not being left at home, unsupported, following the death of the family member or friend. This helps to highlight the fact that every situation is different. For a small minority of patients needing EOL care, ED may be the better place to die. A large-scale study in Australia of over 25,000 palliative care patients nearing the end of their lives demonstrated that control of severe pain and other symptoms is better for those that choose to die in hospital compared to those treated at home [34].

The need for better management of patients within the community, including staff trained in and responsive to the needs of palliative care patients, thus preventing admission to ED, was discussed within two themes. Several reasons were suggested for patients going to ED. A serious deterioration in their medical condition was viewed by a few participants as a legitimate reason for a patient being brought to ED, especially out of hours, when it was not always possible to contact the community team responsible. Patients and families not knowing who to contact for help in the community or being unable to contact their GP, were also stated as reasons why patients with palliative care needs attend ED. Some participants were clearly frustrated by what they considered were inappropriate and sometimes repeated referrals, for example by GPs, care homes and hospices. Several studies [31, 32] have shown that GPs may lack confidence in making an ACP, providing palliative and end-of-life care at home including symptom control especially in non-cancer patients and communicating with families. Having specialist community palliative care services, with lack of clarity over roles of generalist verses specialist services, can further undermine confidence [35]. Several participants in REPLICA discussed the advantage of having clear written guidance for carers to help prevent patients coming into ED unnecessarily (including from hospices and care homes).

The disparity between the availability of support services for ED clinicians during ‘normal’ working hours (which varies, depending on the service) and at other times was brought up within several themes. For example, difficulties in accessing community support outside of hours was discussed as a key factor when deciding whether patients should be admitted to a ward, rather than discharged home from the ED. Participants were satisfied with the support received during normal working hours from the palliative care, oncology, and other medical teams within the Trust. It was felt that fewer patients with palliative care needs come to ED during the day due to support being available elsewhere. However, the challenges and frustrations experienced by ED staff when seeking advice at other times, including from the local hospice, were highlighted by several participants. Participants described problems such as unanswered calls and advice on the treatment of patients not being given when requested. Other studies have highlighted challenges in accessing services outside of normal working hours [30, 33].

Doctors discussed the importance of considering patients’ wishes regarding the preferred treatment, place of treatment and death, as expressed in other studies with clinicians working in ED [36]. It was felt that decisions could be made more easily by medical staff when patients already have a completed ReSPECT form or have an ACP in place (see Eli et al. (2021) for detail on the ReSPECT form) [37].The need to have ‘that conversation’ about dying outside of ED, whilst the patient is still able to do so, was repeatedly raised. Going through the ReSPECT process or deciding on an ACP entails a discussion between the patient, family (where appropriate), and clinicians (nurse or doctor) and results in a ReSPECT form being completed by a clinician on the patient’s preferences for treatment, as well as documenting other helpful information. Published research on the use of ReSPECT forms is limited. Early research showed low rates of completion, with some parts of forms incomplete [37]. The findings from later studies have been more encouraging. A study carried out by the Clinical Outcomes Group Forth Valley showed high rates of form completion and satisfaction of patients and families [38]. It was also found that patients with a completed ReSPECT form were less likely to be admitted to hospital and to die in hospital.

Although in the absence of a written plan, participants recognised the importance of discussing the way forward with the patient and family. This may not be possible in situations where there are time constraints, a patient is unable to communicate and/or the family is unaware of their wishes. In these cases, patients may be given an array of tests and procedures including CPR, even if close to death. Although all the doctors interviewed were prepared to have ‘that conversation’ about dying when appropriate, and said they do so on a daily basis, they felt strongly that ED is not the right place, especially when the patient is too unwell to think rationally about their options and the family is distressed.

### Limitations

Although the NHS HES dataset has been shown to be valid for research purposes, there are a number of limitations [39]. In a large HES dataset, such as the one used in this study, the standard of coding between hospitals may vary resulting in inaccurate and incomplete data or duplications [40]. With respect to the present study, several factors contributed to patients with palliative care needs being underrepresented. Only data for patients who had been recognised as needing palliative care and therefore specifically given the palliative care code (Z51.5) were included in the analysis. Other patients requiring palliative care with a different code, reflecting the medical speciality providing treatment, would not have been included in the analysis. Palliative care is coded as a supportive care service, leading to under-reporting and underutilisation. Patients with an incomplete pathway (i.e. with data collected prior to Year 1 and outcomes from Year 4) were omitted from this study.

The interviews were conducted just prior to the start of the COVID-19 epidemic in the UK, when the focus of research in the Trust where data were collected changed to COVID-19. Hence there was a gap between data collection and completing the study. Interview participants in REPLICA were only from one large hospital trust. However, the HES data illustrates similarities in terms of palliative care admissions to the rest of England. It is likely that the issues raised by participants and the findings can be transferred to other similar, large trusts. Ideally community and hospital staff working with patients with palliative care needs should have also been interviewed to gain their perspectives. This would have helped to build a better picture on communication between the different internal and external agencies. The data were collected at the end of 2019. Another limitation was being unable to work on the analysis until after the worst of the COVID pandemic. However, following the analysis and interpretation of the findings, a meeting was held with members of the hospital and community palliative care teams to discuss how services had changed. The palliative care consultants also read the manuscript prior to finalisation and felt the findings still held value for them.

### The way forward

It was suggested that short education sessions on caring for patients with palliative and EOL care needs within the ED environment, as well as the resources, processes, and pathways they need to be aware of, will help to improve care. Enhancing the palliative care component of training courses of health professionals, and community staff, including GPs, would also be of benefit, although curricula are often overcrowded.

The need for other health professionals, patients and family members to talk about death and dying is a wider societal issue, where people are reluctant to discuss this inevitable life event. However, the need for health professionals working outside of ED to use the ReSPECT form as a basis of “that discussion” was strongly expressed by a number of participants. This particular finding serves to highlight the need for better communication between providers of healthcare, including those working in ED and other specialities within the hospital, community teams and hospices.

Participants agreed that we need to prevent the majority of patients with palliative care needs from attending hospital. We call for a roundtable discussion between palliative care teams, and representatives from community and social services, hospices, the emergency department, patient and public involvement groups and out of hours services, to design a co-ordinated and dedicated service to support patients with palliative care needs in the community, with a clear action plan to ensure that changes to the current service are implemented and reviewed. As Taylor et al. (2022) point out in their scoping review of initiatives to reduce inappropriate or non-beneficial hospital admissions and bed days in people nearing the end of their life, there is much innovation but limited supporting evidence [41]. Therefore, it is imperative that interventions designed to better support palliative care patients and staff caring for these patients, are fully evaluated.

## Conclusion

This study demonstrates that large numbers of palliative care patients are admitted through ED for care. Issues such as out-of-hours care provision and the need to treat patients at home rather than in ED, identified in previous studies of patients and their home carers, are still important. Although the senior clinicians interviewed felt confident about treating palliative patients, uncertainty about the different care pathways and resources available was expressed by most participants. The Emergency Department is not the ideal place for EOL care for most patients, but it is where many will spend some or all of their last hours of life. Further training and resources to support ED staff are therefore needed to ensure those patients have better deaths.

### Electronic supplementary material

Below is the link to the electronic supplementary material.

Supplementary Material 1

## Data Availability

The primary data that support Part 1 of this study were from Hospital Episodes Statistics (HES) and were used under contract and agreement for the current study and so not publicly available. These primary data were deleted one year after receipt through signed declaration as per contract. The analyses are available from Jaimini Rajani – Jaimini rajani@uhcw.nhs.uk, upon reasonable request. The interview data that support Part 2 of the study are available from Dr Jane Sausman, email: Jane.Sausman@uhcw.nhs.uk, upon reasonable request.
