# Supplementary material for: Healthcare professionals’ perspectives of the management of people with palliative care needs in the emergency department of a UK hospital
Source: BMC Palliat Care. 2023 Sep 6;22:129. doi: 10.1186/s12904-023-01248-8 (PMC10481573; doi:10.1186/s12904-023-01248-8)
Supplement: Supplementary file 1 — Supplementary Material 1 [file 12904_2023_1248_MOESM1_ESM.docx]

**Supplementary File 1**

**REPLICA Study Interview Topic Guide**

*The interview will explore the following four topics with the participants. Example questions are provided, but will be adapted to suit the interviewee, their role, and the circumstances of the interview. Probes are also suggested, which may be used as appropriate.*

**Basic demographic questions**

- **Age, gender, job title, length of time working in the Emergency Department (ED)**
- **Ask the participant not to disclose any personal identifiable information about themselves or patients**

**Interview Topics**

1. **Clinician’s understanding and perceptions of palliative care in the ED**

- **Can you tell me what palliative and end of life (EOL) care means to you?**
- **Probes: Definitions of Palliative Care and EOL – can they differentiate**
- **Do you associate the terms with any particular condition/ If yes, can you explain why (or elaborate on your response)?**

**Can you tell me about any training or background you might have in palliative or end of life care?**

**Think about the patients that present to the ED. How do you feel about caring for those with EOL (including dying patients) or Palliative Care needs?**

**Probes: Is this the right place for them?**

1. **Palliative care processes in the ED (general discussion)**

- **Tell me about your role in the ED**
- **Does your role involve recognising or assessing a patient with palliative and EOL care needs?**
- **Are there any tools, guides/forms you might use? If so, can you tell me about these?**
- **Probes: SPICT tool, Advanced care plan, DNACPR forms, Palliative care alert (CRRS)**
- **Show SPICT tool – what are their thoughts re this?**
- **How could it be used in ED?**
- **Any barriers/enablers/benefits?**
- **Can you tell me about the process you go through when deciding whether to admit a patient with palliative care needs?**
- **What sort of things do you consider (and document)?**
  - **Are you aware of alternative emergency pathways (other departments/wards/services) where patients could be referred to from the ED?**
  - **If so, please tell me about these and referring patients**
- **Probes: Acute oncology, rapid response (community), Hospice**
- **Have you had any experiences or interactions with other care providers (e.g hospital/community palliative care team)? If so, can you tell me about these?**
- **Can you tell me what happens when you choose not to admit a patient from ED with palliative care needs?**
- **Would you initiate any follow-up or contact with G.P/community services?**
  - **How might your actions be different, if at all, if you have seen this patient multiple times recently?**
  - **How does this differ if the palliative needs are ‘new’ (i.e not previously known to the patient or their regular care team)?**
- **What can you tell me about how palliative care patients are identified or coded?**
- **E.g the CRRS Palliative Care ‘SPICT’ Alert**

**3. (Anonymised or hypothetical) Case discussions**

- **Can you tell me about a patient with known palliative care needs that you cared for?** *Please keep the case anonymous, or if you prefer, draw on your experiences to describe a hypothetical case.*
- **Have you ever assessed a patient and spotted unmet palliative care needs? If so, could you tell me what happened?**
- **How would you feel about initiating the first conversation about palliative care in this scenario? If you would not wish to do it – why not?**

**4. What could or should be different?**

- **Reflecting on your experiences, why do you think patients with EOL and palliative care needs come to ED?**
- **Do you think more could or should be done to detect or assess patient’s palliative care needs in ED or do you think the present system is adequate?**
  - **If yes, please elaborate on what could be done**
- **What do you think could be done to help support you to manage palliative care needs in the ED?**
- **Is there anything else you would like to add to what we have discussed?**

Thank you.

Would they like to be sent a certificate for revalidation?
